# Role of Hypoxia Inducing Factor-1β in Alcohol-Induced Autophagy, Steatosis and Liver Injury in Mice

**DOI:** 10.1371/journal.pone.0115849

**Published:** 2014-12-23

**Authors:** Hong-Min Ni, Amar Bhakta, Shaogui Wang, Zhenrui Li, Sharon Manley, Heqing Huang, Bryan Copple, Wen-Xing Ding

**Affiliations:** 1 Department of Pharmacology, Toxicology and Therapeutics, The University of Kansas Medical Center, Kansas City, Kansas, United States of America; 2 Laboratory of Pharmacology and Toxicology, School of Pharmaceutical Sciences, Sun Yat-sen University, Guangzhou, China; RWTH Aachen, Germany

## Abstract

Chronic alcohol causes liver hypoxia and steatosis, which eventually develops into alcoholic liver disease (ALD). While it has been known that alcohol consumption activates hepatic hypoxia inducing factor-1α (HIF-1α), conflicting results regarding the role of HIF-1α in alcohol-induced liver injury and steatosis in mice have been reported. In the present study, we aimed to use hepatocyte-specific HIF-1β knockout mice to eliminate the possible compensatory effects of the single knockout of the 1α subunit of HIF to study the role of HIFs in ALD. C57BL/6 wild type mice were treated with acute ethanol to mimic human binge drinking. Matched wild-type and hepatocyte specific HIF-1β knockout mice were also subjected to a recently established Gao-binge alcohol model to mimic chronic plus binge conditions, which is quite common in human alcoholics. We found that acute alcohol treatment increased BNIP3 and BNIP3L/NIX expression in primary cultured hepatocytes and in mouse livers, suggesting that HIF may be activated in these models. We further found that hepatocyte-specific HIF-1β knockout mice developed less steatosis and liver injury following the Gao-binge model or acute ethanol treatment compared with their matched wild type mice. Mechanistically, protection against Gao-binge treatment-induced steatosis and liver injury was likely associated with increased FoxO3a activation and subsequent induction of autophagy in hepatocyte-specific HIF-1β knockout mice.

## Introduction

Alcoholic liver disease (ALD) is a major liver disease, which is a significant health problem in the United States and around the world. The pathogenesis of ALD in humans is characterized by steatosis, which is caused by increased uptake of fat into the liver, increased fatty acid and triglyceride synthesis, and decreased fatty acid oxidation. Approximately 8–20% of heavy drinkers with steatosis progress further to steatohepatitis, fibrosis and cirrhosis, and small percentage of these (3–10%) eventually develop hepatocellular carcinoma (HCC) [1], [2]. Hepatic steatosis is one of the earliest and most common signs of alcohol consumption and is an important step in the progression to ALD. Chronic alcohol consumption also induces apoptotic and necrotic cell death, which also contribute to the pathogenesis of ALD. However, the mechanisms regulating alcoholic steatosis and cell death are poorly understood. Therefore, an understanding of the mechanism by which alcohol leads to excess triglycerides and cell death can possibly lead to novel interventional approaches to prevent or treat ALD.

It is well known that chronic alcohol drinking increases hepatic oxygen consumption resulting in pericentral hypoxia in the liver [3], [4]. Liver tissues can adapt to hypoxic conditions by activating gene transcription programs that regulate glucose uptake and metabolism, erythropoiesis, angiogenesis, cell death and cell proliferation. One family of transcription factors that is activated in response to hypoxia is hypoxia inducible factors (HIFs). HIFs are transcription factors composed of an oxygen-sensitive α subunit and an oxygen insensitive constitutively expressed β subunit. There are three HIF-α subunits (HIF1-α, HIF-2α and HIF-3α), which are all expressed in the liver. Under normoxic conditions, the α subunit is hydroxylated at specific proline residues, which targets it for ubiquitination by the von Hippel-lindau (VHL) tumor suppressor. Ubiquitination then targets it for proteasomal degradation. When oxygen concentration is low, the α subunit is stabilized and translocates to the nucleus where it dimerizes with HIF-1β and regulates expression of target genes. Deletion of VHL in mice leads to severe hepatic steatosis due to the constitutive activation of HIF-2α but not HIF-1α, suggesting HIF-2α plays a more prominent role in regulating hepatic lipid metabolism [5]. Increased hepatic HIF-1α and HIF-2α expression has been found in both acute binge and chronic ethanol feeding in mice. However, conflicting results regarding the role of HIF-1α in alcohol-induced liver injury and steatosis in mice have been reported [6], [7], [8]. Nishiyama et al. [6] reported that hepatocyte-specific HIF-1α knockout mice had more severe liver injury and steatosis than wild type mice after exposing mice to a 6% ethanol-containing liquid diet for 4 weeks. In contrast, Nath et al. [7] reported that hepatocyte-specific HIF-1α knockout mice were protected against steatosis and liver injury after exposure of mice to a similar liquid ethanol diet for 4 weeks (5% ethanol was used in this study). The reasons for these conflicting results are not clear although it has been suggested that differences in housing environments may have contributed [8]. Moreover, it has been reported that stable knockdown of HIF-1α can lead to a compensatory increase in HIF-2α expression. Vice versa, knockdown of HIF-2α can lead to a compensatory increase in HIF-1α expression in HepG2 cells [9]. However, whether or not there was a compensatory increase in HIF-2α or HIF-3α in hepatocyte-specific HIF-1α knockout mice has not been investigated in alcohol feeding studies. Because the α subunit must hetero-dimerize with HIF-1β to enable its transcriptional activation, hepatocyte-specific HIF-1β knockout mice would eliminate the compensatory effects of a single knockout of the α subunit of HIF. This would provide a more suitable model for studying the role of HIFs in ALD.

Autophagy is a conserved catabolic process that degrades cellular proteins and damaged/excess organelles such as the mitochondria (mitophagy) and lipid droplets (lipophagy). We and others have recently demonstrated that activation of autophagy protects against alcohol-induced steatosis and liver injury in mice and cultured hepatocytes [10], [11], [12], [13], [14]. In addition to regulating glucose and lipid metabolism, emerging evidence also suggests that hypoxia-induced HIF activation can also regulate autophagy. HIF activation induced-autophagy could be mediated by the up-regulation of two atypical BH3-only Bcl-2 family proteins, the Bcl-2/E1B 19 kDa-interacting protein 3 (BNIP3) and BCL2/adenovirus E1B interacting protein 3-like (BNIP3L/NIX, hereafter referred to as NIX). It has been proposed that BNIP3 and NIX can induce autophagy by direct interaction with Bcl-2/Bcl-X_L_ which in turn releases Beclin-1 from the Bcl-2/Bcl-X_L_ complex to enhance autophagy [15], [16]. However, it is not known whether alcohol-induced HIF activation also contributes to autophagy.

In the present study, we examined the role of HIF-1β in alcohol-induced liver injury using primary cultured hepatocytes treated with ethanol. In addition, wild type and hepatocyte-specific HIF-1β knockout mice were treated with either acute ethanol to mimic human binge drinking or a recently developed Gao-binge model to mimic chronic plus binge drinking conditions, which is often found in human alcoholics [17]. The Gao-binge model has been shown to increase liver injury and inflammation which is not observed in other alcohol animal models. We found that ethanol treatment increased BNIP3 and NIX expression in primary cultured hepatocytes, which was dependent on HIF-1β. Intriguingly, while ethanol administration also increased the expression of BNIP3 and NIX in wild type mouse livers, hepatocytes-specific deletion of HIF-1β did not inhibit ethanol-induced expression of BNIP3 and NIX. This was likely due to a compensatory activation of forkhead box O3a (FoxO3a), another important transcription factor that regulates expression of BNIP3 and NIX, as well as other autophagy-related genes. Consequently, hepatocyte-specific HIF-1β knockout mice had less steatosis and liver injury following Gao-binge treatment.

## Materials and Methods

### Reagents

Antibodies used in this study were BNIP3 (#Ab10433), NIX (#Ab8399) and cyp2E1 (#Ab19140) from Abcam, p62 (#H00008878-M01) from Abnova, FoxO3 (#2497), phosphorylated Akt (S473, #4060), Akt (#2966), phosphorylated 4EBP1 (Ser65, #9451), 4EBP1 (#9452) and Lamin A/C (#2032) from Cell Signaling Biotechnology, β-actin (#A5441) from Sigma-Aldrich, MnSOD (#sc-30080) from Santa Cruz Biotechnology, and horseradish peroxidase–conjugated secondary antibody from Jackson ImmunoResearch Laboratory. The rabbit polyclonal anti-LC3B antibody was generated as described previously [10]. Adenovirus GFP-LC3 was generated as described previously [10]. Chloroquine (CQ) was from Sigma-Aldrich. Ethanol was from Pharmaco, Inc. and all other chemicals were from Sigma, Invitrogen, or Calbiochem.

### Animal experiments

C57BL/6 mice and Albumin Cre mice were obtained from The Jackson Laboratory. HIF-1β Flox/Flox mice were obtained from Dr. Frank Gonzalez at NIH [18]. Albumin Cre negative (HIF-1β, Alb Cre-) and HIF-1β Flox/Flox, Albumin Cre positive (HIF-1β, Alb Cre+) mice were maintained in a C57BL/6 background. All mice received care in compliance with the protocols approved by the Institutional Animal Use and Care Committee of the University of Kansas Medical Center. Acute ethanol treatment was performed as described by us previously [10], [19]. This model was designed to achieve blood alcohol levels, behavioral effects, and physiological effects comparable to human binge drinking. Briefly, after 6 hours of fasting, male C57BL/6 wild type mice, HIF-1β, Alb Cre- and HIF-1β, Alb Cre+ mice were administrated 33% (v/v) ethanol at a total accumulative dose of 4.5 g/kg body weight by four equally divided gavages in 15-minute intervals. Control mice received the same volume of water. After 6, 12 and 16 hours of treatment, the mice were sacrificed, and blood samples and liver tissues were collected. In some experiments, mice were sacrificed after 16 hours of treatment. Gao-binge (Chronic+binge) treatment was performed as described previously [17]. Briefly, male HIF-1β, Alb Cre- (wild type) and HIF-1β, Alb Cre+ (hepatocyte-specific knockout) mice were fed a Lieber-DeCarli liquid control diet (Bioserv # F1258SP) for the first 5 days for adaptation. They were then maintained on a 5% ethanol (vol/vol) diet for 10 consecutive days. Consumption was recorded daily and weight was recorded every other day. Isocaloric amounts of non-alcohol containing control diets were given to pair-fed mice. On the morning of the final day, mice were treated with either 4.5 g/kg ethanol (Ethanol diet+ethanol, ED+E) or a 45% (w/v) maltose solution (Control diet+maltose, CD+M) via gavage similar to the acute model. Mice were euthanized 8 hours later, and blood samples and liver tissues were collected. Livers were excised and weighed, and portions were frozen in liquid nitrogen for biochemical assays or formalin for histopathological analysis. Liver injury was assessed by determination of serum alanine aminotransferase (ALT) activity as we described previously [20]. Total liver lysates were prepared using RIPA buffer (1% NP40, 0.5% sodium deoxycholate, 0.1% sodium dodecyl (lauryl) sulfate).

### Histopathological Analysis

Frozen sections were prepared from liver samples frozen in OCT media and subjected to Oil Red O staining. Oil Red O (0.5%) solution was prepared in propylene glycol. Slides were placed in a 37°C incubator to air dry and washed with PBS. Slides were then placed in propylene glycol for 3 minutes before being stained in Oil Red O for 10 minutes in a 60°C oven. Slides were destained with propylene glycol and rinsed with distilled water. Finally the slides were stained with hematoxylin for 5 seconds and washed in tap water for 3 minutes. Slides were mounted in aqueous mounting medium followed by microscopy.

### Neutrophil Staining

Neutrophil accumulation in the liver was assessed by staining tissue sections by using the rat anti-mouse DAB detection system (Vector Labs, Burlingame, CA) and antibody against lymphocyte antigen B superfamily (Ly6B) (AbD Serotec Raleigh, NC), a specific surface marker for neutrophils as we described previously [21]. Briefly, slides containing liver sections were deparaffinized, rehydrated, and then incubated with peroxidase suppressor (Sigma, St. Louis, MO) for 30 minutes at room temperature. Primary antibody was used at a dilution of 1∶200. After development with DAB, the tissues were counterstained with hematoxylin. The number of Ly6B positive cells were counted from 10 different fields (40X) in a blinded fashion.

### Triglyceride (TG) analysis

Hepatic TG extraction was performed as described previously [19]. Frozen liver tissues (50–100 mg) were powdered using a mortar and pestle. One mL of a chloroform-methanol mixture (2∶1) was added to the liver powder and incubated at room temp for 1 hour with shaking. Then 200 µL of ddH_2_O was added to the sample and centrifuged for 5 minutes at 3000 g. The lower lipid phase was collected and dried, and the pellet was redissolved in Tert-Butanol-Triton X-114-methanol mix (2∶3). TG analysis was carried out using a commercially available kit (Pointe Scientific, Inc. GPO-Triglyceride Reagent Set) following the manufacturer's instructions.

### Primary hepatocyte culture

Murine hepatocytes were isolated by using a retrograde, nonrecirculating perfusion system with 0.05% Collagenase Type IV (Sigma) as we described previously [10]. Cells were cultured in William's medium E containing 10% fetal bovine serum for 2 hours to allow attachment. Cells were then cultured in the same medium without serum overnight before treatment. All cells were maintained in a 37°C incubator with 5% CO_2_.

### Nuclear fractionation

Liver nuclear fractionation was performed using NE-PER nuclear and cytoplasmic extraction reagents (Thermo Fisher) as we described previously [19]. Briefly, liver tissues were cut into small pieces, homogenized in cytoplasmic extraction reagent I followed by the addition of cytoplasmic extraction reagent II. The samples were then centrifuged. The supernatants contained the cytoplasmic fraction, and the insoluble pellets contained the nuclear fraction which was resuspended in nuclear extraction reagent.

### Immunoblot assay

Cells were washed with PBS and lysed in RIPA buffer. Twenty micrograms of protein from each sample was separated by SDS-PAGE and transferred to PVDF membranes. The membranes were stained with primary antibodies followed by secondary horseradish peroxidase-conjugated antibodies. The membranes were further developed with SuperSignal West Pico chemiluminescent substrate (Thermo Fisher). Densitometry was performed using ImageJ software. The resulting bands were normalized using beta-actin (total lysates) or Lamin A/C (nuclear extracts), and expressed as means ± SEM.

### Electron Microscopy (EM)

Liver tissues were fixed with 2.5% glutaraldehyde in 0.2 M sodium cacodylate buffer (pH 7.4), followed by 1% OsO_4_. After dehydration, thin sections were stained with uranyl acetate and lead citrate for observation under a JEM 1011CX electron microscope (JEOL). Images were acquired digitally. The average number of autophagosomes from each cell was determined from a randomly selected pool of 15 to 20 fields under each condition.

### Fluorescence Microscopy for autophagy

To examine autophagy, primary hepatocytes were seeded in a 12 well-plate (2×105 in each well) and infected with adenovirus-GFP-LC3 (100 viral particles per cell) overnight. Cells were treated with ethanol (80 mM) in the presence or absence CQ (20 µM). After treatment, cells were fixed with 4% paraformaldehyde in phosphate buffered saline (PBS) for 2 hours at room temperature or kept at 4°C for fluorescence microscopy. Fluorescence images were acquired using a Nikon Eclipse 200 fluorescence microscope with MetaMorph software.

### Real-time Polymerase chain reaction

RNA was isolated from mouse livers using Trizol (Invitrogen) and reverse transcribed into cDNA by RevertAid reverse transcriptase (Fermentas) as we described previously [22]. Real-time PCR was used to quantify *atg5, becn-1/atg6, atg7, map1lc3b/LC3B* (Microtubule-associated protein light chain 3B), bnip3, *bnip3L/nix* (BCL2/adenovirus E1B interacting protein 3-like), *foxo3*, *hif-1α*, *hif-2α*, Stimulated with retinoic acid (Stra) 13/differentiated embryo chondrocyte 1 (*dec1*), *dec2*, *gpat1* (glycerol-3-phosphate acyltransferase 1), *scd1* (stearoyl-Coenzyme A desaturase 1), *me3* (malic enzyme 3), *fasn* (fatty acid synthase), *acc1α* (acetyl-Coenzyme A carboxylase alpha), *fdps* (farnesyl diphosphate synthase), *acox1* (acyl-Coenzyme A oxidase 1), *mcad* (acyl-Coenzyme A dehydrogenase, medium chain), *cpt1α* (carnitine palmitoyltransferase 1 alpha), *crot* (Peroxisomal carnitine O-octanoyltransferase), *cd36*, *lipin1*, *lipin1α*, *lipin1β* and β-actin mRNA and performed on a BioRad CTX384 real-time PCR instrument (BioRad) using Universal SYBR qPCR reagent (BioRad). Primer Sequences are listed in Table 1.

**Table 1:** RT-RCR Primers Used in This Study.

β-actin	Forward: 5' - TGTTACCAACTGGGACGACA - 3'
	Reverse: 5' - GGGGTGTTGAAGGTCTCAAA - 3'
Atg5	Forward: 5' - GACCACAAGCAGCTCTGGAT - 3'
	Reverse: 5' - GGTTTCCAGCATTGGCTCTA - 3'
Beclin1/Atg6	Forward: 5' - TGATCCAGGAGCTGGAAGAT - 3'
	Reverse: 5' - CAAGCGACCCAGTCTGAAAT - 3'
Atg7	Forward: 5' - TCCGTTGAAGTCCTCTGCTT - 3'
	Reverse: 5' - CCACTGAGGTTCACCATCCT - 3'
LC3B	Forward: 5' - CCGAGAAGACCTTCAAGCAG - 3'
	Reverse: 5' - ACACTTCGGAGATGGGAGTG - 3'
Bnip3	Forward: 5' - AGCTTTGGCGAGAAAAACAG - 3'
	Reverse: 5' - TCCAATGTAGATCCCCAAGC - 3'
Bnip3L	Forward: 5' - AACAACAACTGCGAGGAAGG - 3'
	Reverse: 5' - GTCCCTGCTGGTATGCATCT - 3'
FoxO3	Forward: 5' - AGCCGTGTACTGTGGAGCTT - 3'
	Reverse: 5' - TCTTGGCGGTATATGGGAAG - 3'
HIF1α	Forward: 5' - TCAAGTCAGCAACGTGGAAG - 3'
	Reverse: 5' - TATCGAGGCTGTGTCGACTG - 3'
HIF2α	Forward: 5' - AGCCAAACACGGAGGATATG - 3'
	Reverse: 5' - GTGTGGCTTGAACAGGGATT - 3'
DEC1	Forward: 5' - GGATTTTGCCCACATGTACC - 3'
	Reverse: 5' - TCAATGCTTTCACGTGCTTC - 3'
DEC2	Forward: 5' - GCTTGAAGCGAGACGATACC - 3'
	Reverse: 5' - GGCTGTTAGCGCTTTCAAGT - 3'
GPAT1	Forward: 5' - AGCAAGTCCTGCGCTATCAT - 3'
	Reverse: 5' - CTCGTGTGGGTGATTGTGAC - 3'
SCD1	Forward: 5' - TGCGATACACTCTGGTGCTC - 3'
	Reverse: 5' - TAGTCGAAGGGGAAGGTGTG - 3'
ME3	Forward: 5' - AGATGTTTGCCCAAGACCAC - 3'
	Reverse: 5' - GCTCAGGGCAAAGACGATAG - 3'
FASn	Forward: 5' - TGGGTTCTAGCCAGCAGAGT - 3'
	Reverse: 5' - ACCACCAGAGACCGTTATGC - 3'
ACC1α	Forward: 5' - CTCCAGGACAGCACAGATCA - 3'
	Reverse: 5' - TGACTGCCGAAACATCTCTG - 3'
FDPS	Forward: 5' - ATGGAGATGGGCGAGTTCTTC - 3'
	Reverse: 5' - CCGACCTTTCCCGTCACA - 3'
AcoX1	Forward: 5' - CAGGAAGAGCAAGGAAGTGG - 3'
	Reverse: 5' - CCTTTCTGGCTGATCCCATA - 3'
MCAD	Forward: 5' - AGGTTTCAAGATCGCAATGG - 3'
	Reverse: 5' - CTCCTTGGTGCTCCACTAGC - 3'
CPT1α	Forward: 5' - CCAGGCTACAGTGGGACATT - 3'
	Reverse: 5' - GAACTTGCCCATGTCCTTGT - 3'
Crot	Forward: 5' - TACTTTTACCACGGCCGAAC - 3'
	Reverse: 5' - GACGGTCAAATCCTTTTCCA - 3'
CD36	Forward: 5' - ATGGGCTGTGATCGGAACTG - 3'
	Reverse: 5' - AGCCAGGACTGCACCAATAAC - 3'
LIPIN1	Forward: 5' - CCCTCGATTTCAACGTACCC - 3'
	Reverse: 5' - GCAGCCTGTGGCAATTCA - 3'
LIPIN1α	Forward: 5' - GGTCCCCCAGCCCCAGTCCTT - 3'
	Reverse: 5' - GCAGCCTGTGGCAATTCA - 3'
LIPIN1β	Forward: 5' - CAGCCTGGTAGATTGCCAGA - 3'
	Reverse: 5' - GCAGCCTGTGGCAATTCA - 3'
IL-6	Forward: 5' - ACAAGTCGGAGGCTTAATTACACAT - 3'
	Reverse: 5' - TTGCCATTGCACAACTCTTTTC - 3'
TNFα	Forward: 5' - CGTCAGCCGATTTGCTATCT - 3'
	Reverse: 5' - CGGACTCCGCAAAGTCTAAG - 3'
Mip1a	Forward: 5' - TGAGAGTCTTGGAGGCAGCGA - 3'
	Reverse: 5' - TGTGGCTACTTGGCAGCAAACA - 3'
Mip1b	Forward: 5' – AACACCATGAAGCTCTGCGT - 3'
	Reverse: 5' - AGAAACAGCAGGAAGTGGGA - 3'
Mip2	Forward: 5' – CTCAGAGGAAGACGATGAAG - 3'
	Reverse: 5' - GACGAGTTATCCCAGCCAAA - 3'

### Statistical Analysis

Experimental data were subjected to Student t-test or one way analysis of variance analysis with Scheffé's post hoc test where appropriate. P<0.05 was considered significant.

## Results

### Acute alcohol treatment increases expression of BNIP3 and NIX in primary mouse hepatocytes and in mouse liver

**Figure 1:**
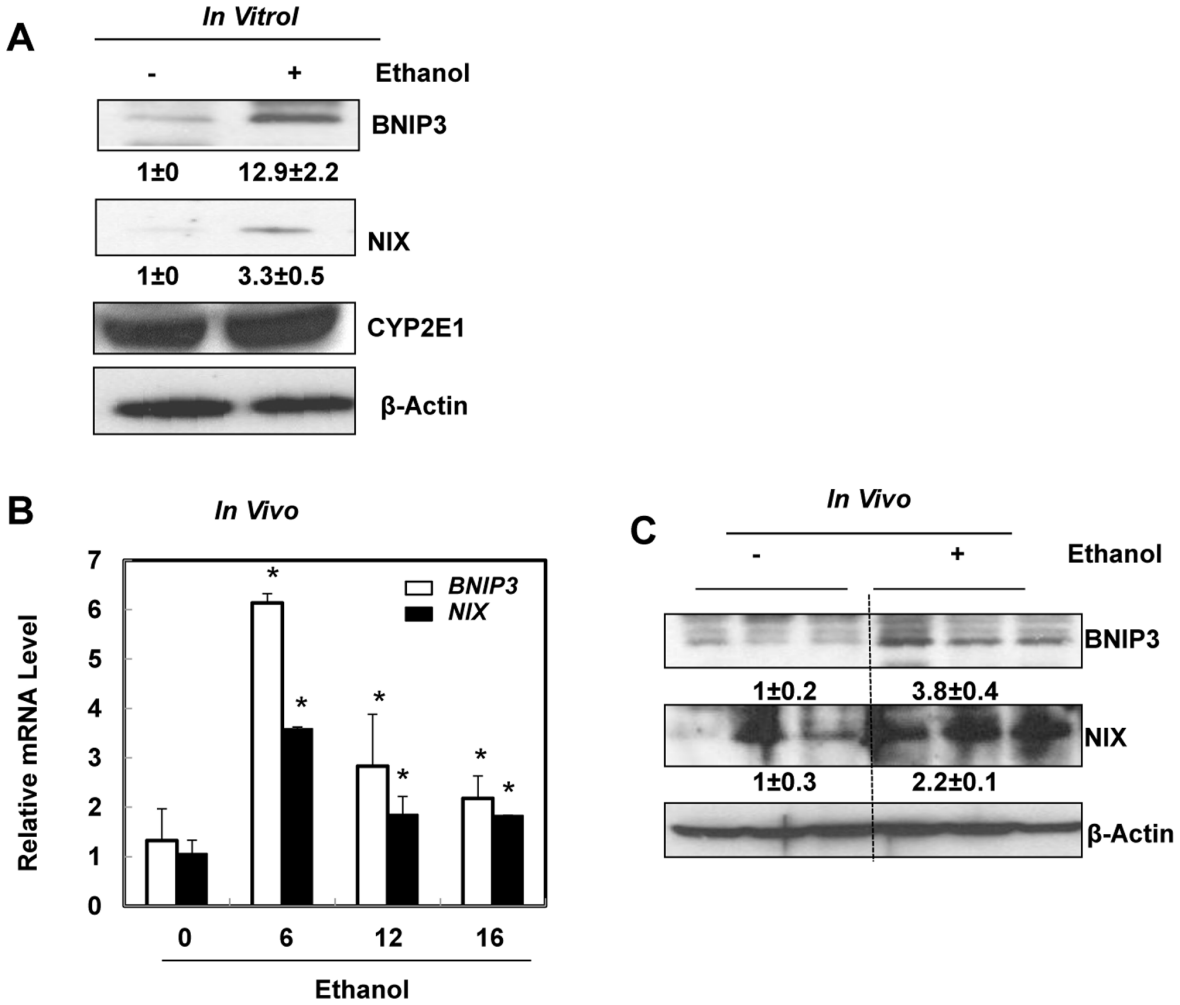
Acute ethanol treatment increased expression of BNIP3 and NIX in primary hepatocytes and mouse liver. Primary cultured mouse hepatocytes were treated with ethanol (80 mM) for 6 hours. Densitometry was performed and data are presented as a ratio vs control (n = 3). (**A**) Total cell lysates from cultured hepatocytes were subjected to western blot analysis. (**B**) Male C57BL/6 mice were treated with either water or ethanol (4.5 g/kg) by gavage for 6, 12 or 16 hours. Hepatic mRNA was isolated and real-time RT-PCR was performed as described in the Materials and Methods. Data are presented as means ± SE (n = 4–6). * p<0.05, vs 0 hour control. One way ANOVA with Scheffé's post hoc test. (**C**) Mice were treated as in (**B**) and total liver lysates from the mice (16 hours treatment) were subjected to western blot analysis. Densitometry was performed and data are presented as a ratio of control (n = 3).

We previous showed that ethanol treatment activates FoxO3a in cultured mouse hepatocytes and increases mRNA levels of BNIP3 and NIX [19]. We first examined the protein levels of BNIP3 and NIX after ethanol treatment in primary cultured mouse hepatocytes. Ethanol treatment increased BNIP3 and NIX proteins in primary cultured mouse hepatocytes (**Fig. 1A**). As expected, ethanol also increased CYP2E1 expression in primary hepatocytes. In line with the *in vitro* findings, acute ethanol treatment also increased mRNA levels of BNIP3 and NIX in a time-dependent fashion in C57BL/6 wild type mouse livers (**Fig. 1B**). BNIP3 and NIX protein were also increased in the liver after acute ethanol treatment in mice (**Fig. 1C**). These results indicate that ethanol increases BNIP3 and NIX levels both *in vitro* (hepatocytes) and *in vivo* (mouse livers).

### Ethanol-induced upregulation of BNIP3 and NIX in primary mouse hepatocytes was dependent upon HIF-1β

**Figure 2:**
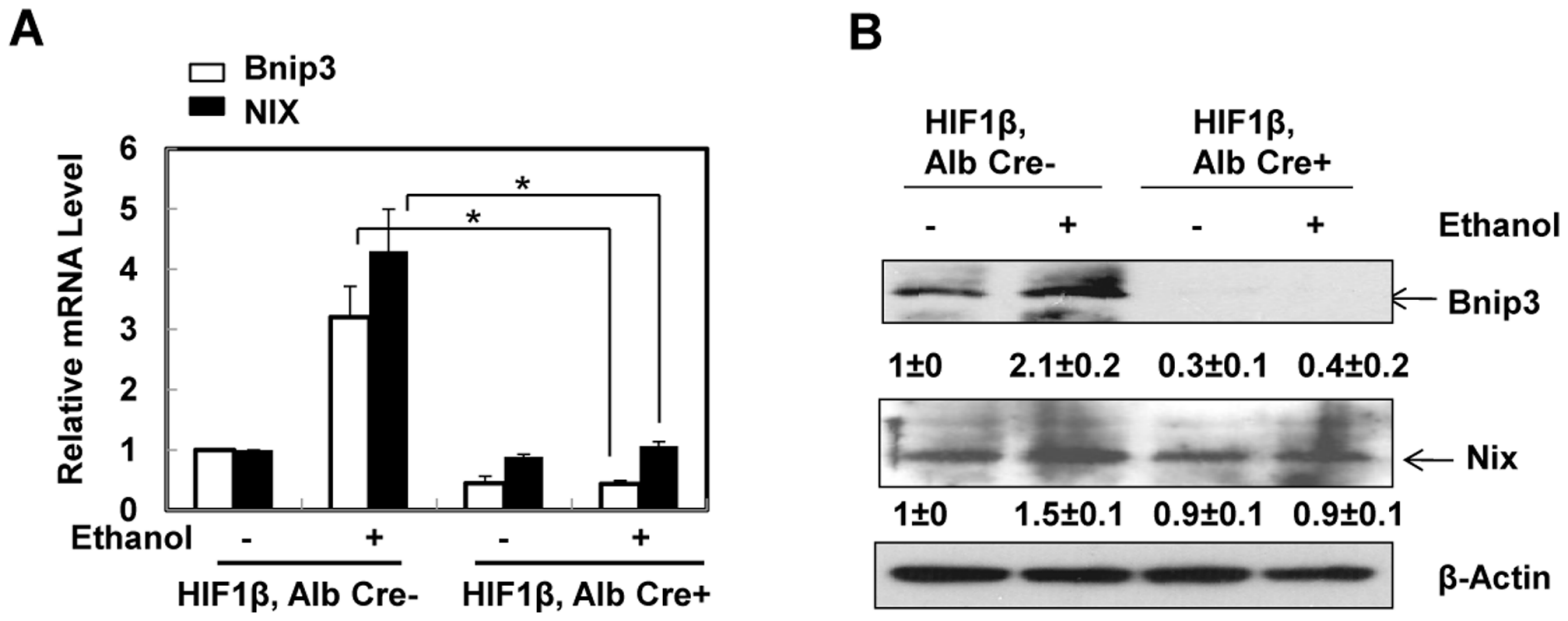
Ethanol-induced expression of BNIP3 and NIX was abolished in primary cultured HIF-1β knockout mouse hepatocytes. (**A**) Primary cultured wild type and HIF-1β knockout hepatocytes were treated with ethanol (80 mM) for 6 hours. mRNA was isolated from cultured hepatocytes and real-time RT-PCR was performed as described in the Materials and Methods. Data are presented as means ± SE (n = 4). * p<0.05. One way ANOVA with Scheffé's post hoc test. (**B**) Cells were treated as in (**A**), total cell lysates were subjected to western blot analysis. Densitometry was performed and data are presented as a ratio vs HIF-1β, Alb Cre- control (n = 3).

We next determined whether HIF-1β was required for ethanol-induced upregulation of BNIP3 and NIX in primary mouse hepatocytes. Ethanol treatment increased mRNA levels of BNIP3 and NIX approximately 3 and 4.5 fold respectively compared to non-treated wild type hepatocytes. In contrast, upregulation of BNIP3 and NIX was prevented in hepatocytes from HIF-1β knockout mice (HIF-1β, albumin Cre+ cells) (**Fig. 2** **A**). Consistent with the mRNA changes, protein levels of BNIP3 were also dramatically reduced in HIF-1β knockout hepatocytes regardless of ethanol treatment (**Fig. 2** **B**). There was no difference for the basal protein levels of NIX between the wild type and HIF-1β knockout hepatocytes, however, alcohol-induced increases in NIX protein were inhibited in HIF-1β knockout hepatocytes. These results indicate that HIF-1β is required for ethanol mediated upregulation of BNIP3 and NIX in primary mouse hepatocytes. Our data also seem to suggest that alcohol-induced changes in BNIP3 are more potent in a HIF-1β dependent manner than that of NIX in primary cultured hepatocytes.

### HIF-1β is dispensable for ethanol-induced autophagy in primary mouse hepatocytes

**Figure 3:**
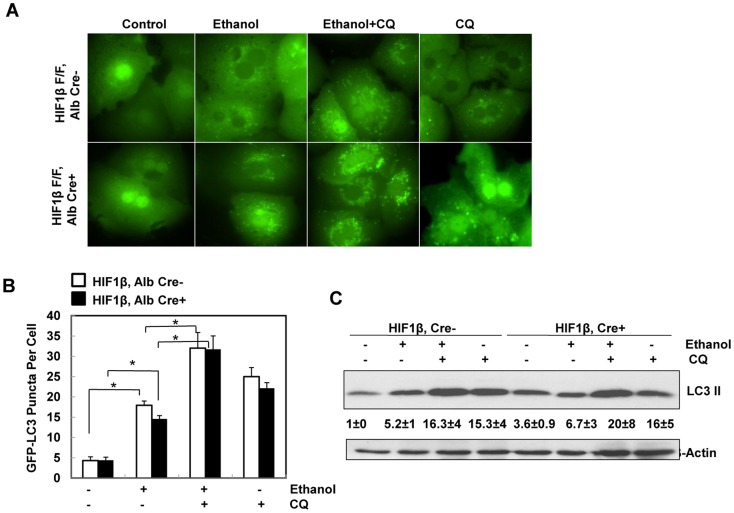
Ethanol induced autophagic flux in primary mouse hepatocytes independent of HIF-1β. Primary cultured wild type and HIF-1β knockout mouse hepatocytes were infected with Ad-GFP-LC3 (100 viral particles per cell) overnight followed by ethanol (80 mM) treatment with or without CQ (20 µM) for 6 hours. (**A**) Representative fluorescence images of GFP-LC3 from each group are shown. (**B**) The number of GFP-LC3 puncta per cell was quantified (more than 20 cells were counted from each independent experiment) and data are presented as means ± SE (n = 3 independent experiments). * p<0.05. One way ANOVA analysis with Scheffé's post hoc test. (**C**) Cells were treated as in (**A**), total cell lysates were subjected to western blot analysis. Densitometry was performed and data are presented as a ratio of HIF-1β, Alb Cre- control (n = 3).

To determine whether HIF-1β is required for ethanol-induced autophagic flux in primary hepatocytes, isolated hepatocytes from HIF-1β, albumin Cre negative (wild type) and HIF-1β, albumin Cre positive (hepatocyte-specific knockout) mice were infected with adenovirus GFP-LC3 overnight and then treated with ethanol (80 mM) in the presence or absence of chloroquine (CQ), a lysosomal inhibitor [23], [24]. Ethanol treatment significantly increased the number of GFP-LC3 puncta in both wild type and HIF-1β knockout hepatocytes (**Figs. 3A & B**), which represent autophagosomes. Combination of CQ further enhanced the number of GFP-LC3 puncta induced by ethanol in both wild type and HIF-1β knockout hepatocytes, suggesting that the ethanol induced autophagic flux in hepatocytes does not require HIF-1β. Immunoblot analysis confirmed the increase in the membrane-associated PE-conjugated form of LC3 (LC3-II) in ethanol-treated mouse hepatocytes, which was further increased in the presence of CQ in wild type hepatocytes (**Fig. 3C**). Ethanol treatment alone only mildly increased LC3-II levels in HIF-1β knockout hepatocytes, which already had a higher basal LC3-II level when compared to wild type hepatocytes. Nevertheless, the LC3-II levels were much higher in the ethanol and CQ co-treatment group when compared to either treatment alone (**Fig. 3C**). These data indicate that ethanol treatment increases autophagic flux in primary hepatocytes, which is independent of HIF-1β. Since HIF-1β knockout hepatocytes had much lower levels of BNIP3 and NIX after ethanol treatment (**Fig. 2**), these data also suggest that BNIP3 and NIX are not required for ethanol-induced autophagy in primary hepatocytes.

### HIF-1β hepatocyte-specific knockout mice had less liver injury and steatosis in response to Gao-binge ethanol treatment

**Figure 4:**
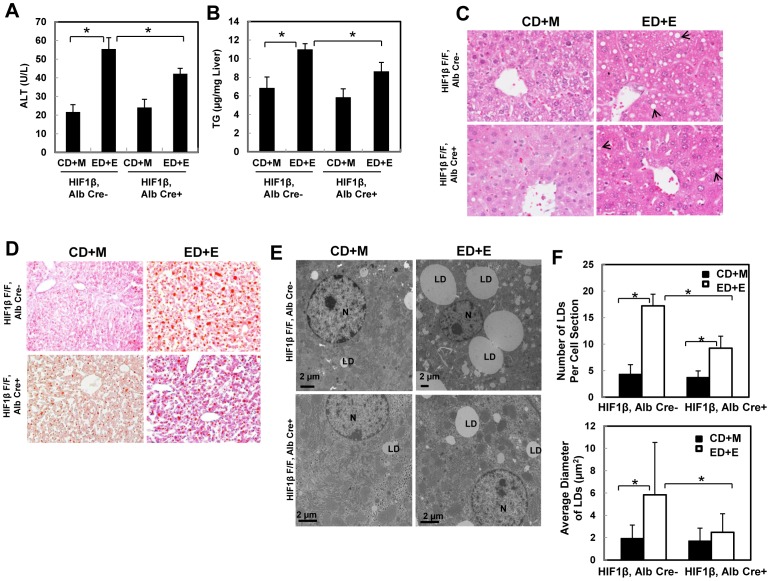
Hepatocyte-specific HIF-1β knockout mice were resistant to steatosis and liver injury from the Gao binge model. Age matched male wild type and hepatocyte-specific HIF-1β knockout mice were subjected to Gao-binge treatment. Serum ALT (**A**) and hepatic TG (**B**) were measured. Data are presented as means ± SE (n = 3–8). * p<0.05. One way ANOVA with Scheffé's post hoc test. Representative photographs of H &E staining (**C**) and Oil O Red staining are shown (**D**). Arrows: hepatic lipid droplets. Representative EM images are shown in (**E**). M: Mitochondria; N: Nuclei; LD: lipid droplet; Bar: 500 nm. The number and size (average diameter) of LDs per cell section was quantified (**F**), and data are presented as means ± SE (more than 20 cell sections and 80 LDs). * p<0.05. One way anova analysis with Scheffé's post hoc test.

We found that the serum ALT levels were significantly elevated in Gao-binge-treated HIF-1β wild type mice. This was diminished in HIF-1β hepatocyte-specific knockout mice (**Fig. 4** **A**). Moreover, hepatic triglyceride levels were also increased in HIF-1β wild type mice however this increase was inhibited in HIF-1β hepatocyte-specific knockout mice (**Fig. 4** **B**). Histological analysis by H & E staining also revealed increased steatosis, as demonstrated by increased lipid vacuoles, in Gao-binge treated HIF-1β wild type mice (arrows, **Fig. 4C**), which was markedly attenuated in HIF-1β hepatocyte-specific knockout mice (**Fig. 4** **C**). Oil Red O staining confirmed these findings and demonstrated a diminished Oil Red O staining in Gao-binge treated HIF-1β hepatocyte-specific knockout mouse livers (**Fig. 4** **D**). Finally, EM studies also revealed a marked increase in the number of hepatic lipid droplets (LD) in Gao-binge treated HIF-1β wild type mice. The diameters of some LDs were greater than the cell nuclei. However, both the size and the number of LD after Gao-binge treatment were diminished in HIF-1β hepatocyte-specific knockout mice compared with wild type mice (**Fig. 4** **E & F**). Collectively, these results indicate that HIF-1β hepatocyte-specific knockout mice are resistant to Gao-binge treatment-induced liver injury and steatosis compared with wild type mice.

### Changes of hepatic lipid metabolic genes after Gao-binge treatment in wild type and HIF-1β hepatocyte-specific knockout mice

**Figure 5:**
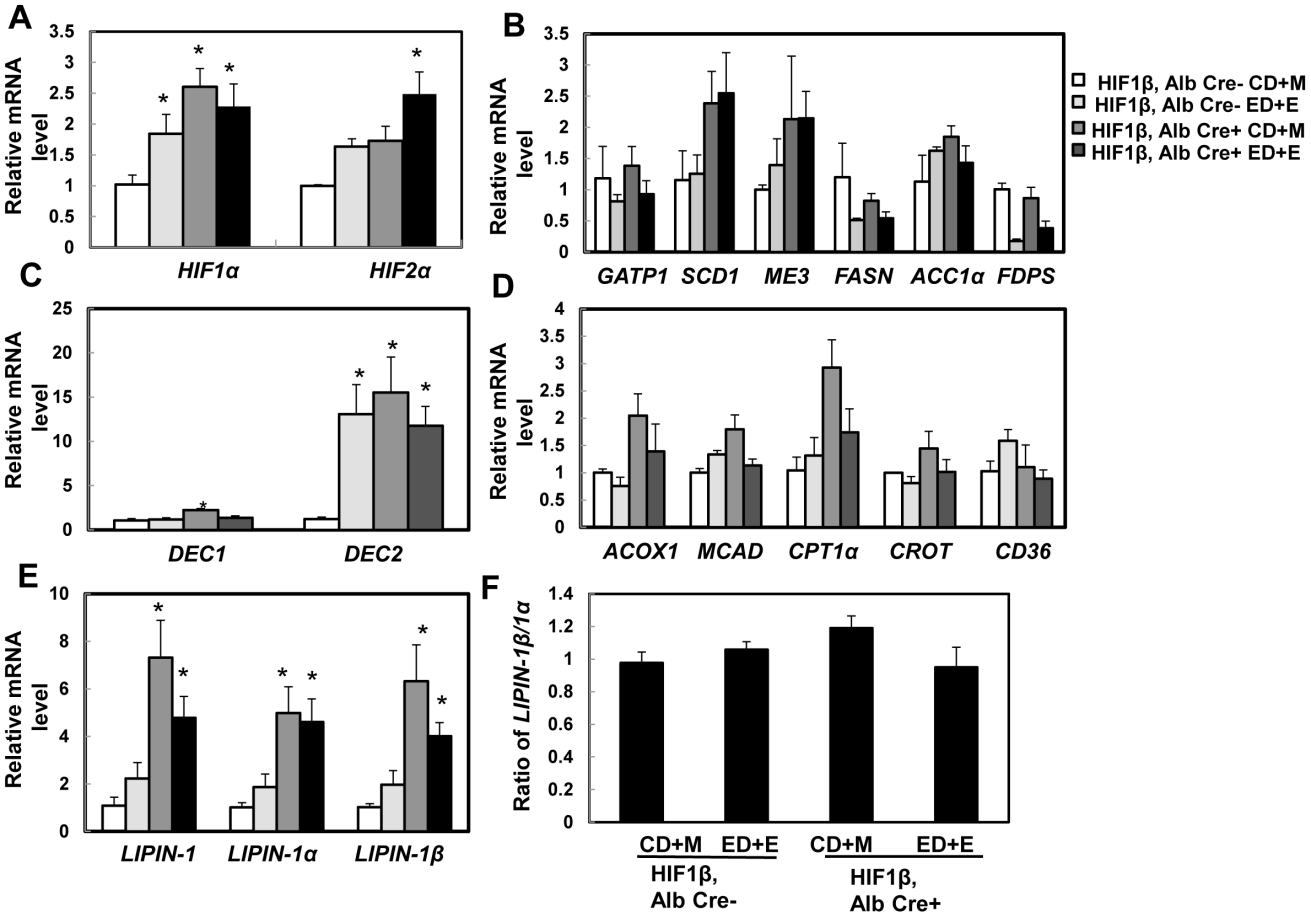
Expression of lipid metabolism genes after Gao-binge treatment in wild type and hepatocyte-specific HIF-1β knockout mice. Age matched male wild type and hepatocyte-specific HIF-1β knockout mice were subjected to Gao-binge treatment. Hepatic mRNA was isolated, and real-time RT-PCR was performed as described in the Materials and Methods to quantify expression of *HIF*(A), lipogenesis genes (B), *DEC1* and *DEC2* (C), fatty acid oxidation (D), and *LIPIN-1* (E). The ratio of *LINPIN-1β/LINPIN-1α* was calculated based on (E) and is shown in (F). Data are presented as means ± SE (n = 3–5). CD+M: Control diet+maltose; ED+E: Ethanol diet+ethanol binge. * p<0.05, vs HIF-1β CD+M group. One way ANOVA with Scheffé's post hoc test.

Chronic ethanol feeding has been shown to induce liver hypoxia and activate HIF [6], [7], [25]. Consistent with these previous findings, we found that Gao-binge treatment increased expression of hepatic *HIF-1α* and *HIF-2α* in wild type mice. Surprisingly, expression of hepatic *HIF-1α* and *HIF-2α* were higher at baseline (1.5 and 0.5 fold increase respectively) and further treatment with Gao-binge did not cause any further significant changes in HIF-1β hepatocyte-specific knockout mice (**Fig. 5A**). These data suggest that the lack of HIF-1β in the mouse liver may cause a compensatory activation of HIF-1α and HIF-2α. It was previously reported that chronic ethanol feeding (4 week Lieber-DeCarli liquid diet) increased the expression of lipogenic genes [6]. We found that Gao-binge treatment had no effects on the expressions of *ME3, ACC1α* and *SCD1* whereas *GPAT1, FASn* and *FDPS* were markedly decreased in wild type mice although only the change of *FDPS* reached statistic difference. Similar to wild-type mice, Gao-binge treatment decreased the expression of *GPAT1, FASn* and *FDPS* in hepatocyte-specific HIF-1β knockout mice although only the change of *FDPS* reached statistical difference. However, hepatocyte-specific HIF-1β knockout mice tend to have increased basal hepatic expression of *SCD1*, *ME3* and *ACC1α*, which seemed to be less affected after Gao-binge treatment (**Fig. 5B**). Stimulated with retinoic acid (Stra) 13/differentiated embryo chondrocyte 1 (DEC1) and 2 (DEC2) are HIF-1 regulated transcriptional repressors that inhibit SREBP-1c expression in response to hypoxia. We found that Gao-binge treatment did not affect the expression of hepatic *DEC1* but markedly increased expression of *DEC2* in wild type mice. Hepatocyte-specific HIF-1β knockout mice had significantly higher basal expression of hepatic *DEC1* and *DEC2*, in particular the basal expression of *DEC2* was almost 15 fold higher than wild type mice. These levels did not change significantly after Gao-binge treatment in hepatocyte-specific HIF-1β knockout mice (**Fig. 5C**). These data suggest that steatosis in Gao-binge-treated mouse livers is less likely due to the increased lipogenesis. The expression levels of genes related to fatty acid oxidation in mitochondria (*CPT1* and *MCAD*) and peroxisomes (*ACOX1*) as well as fatty acid uptake (*CD36*) were less affected by Gao-binge treatment in wild type and hepatocyte-specific HIF-1β knockout mice, except that the basal hepatic expression levels of *CPT1* and *ACOX1* were much higher in hepatocyte-specific HIF-1β knockout mice (increase 2 and 1 fold respectively) (**Fig. 5D**). LIPIN-1 is a protein that has dual roles in lipid biosynthesis and gene expression. The liver LIPIN-1 has two major isoforms, LIPIN-1α and LIPIN-1β, derived from LIPIN1 alternative mRNA splicing [26]. In hepatocytes, LIPIN-1α predominantly localizes in the nuclear and mainly acts as a transcriptional co-activator to promote PGC1α and PPARα-mediated transcription of genes involved in fatty acid oxidation and also acts as a transcriptional repressor for de novo lipogenesis [26]. LIPIN-1β is mainly located in the cytoplasm and functions as an Mg2+-dependent phosphatidic acid phosphohydrolase in the triglyceride synthesis pathway and increased hepatic lipid accumulation [27]. Increased ratio of *LIPIN-1β*/*LIPIN-1α* has been shown to promote chronic ethanol-induced hepatic steatosis [27]. We found Gao-binge treatment increased total hepatic expression of *LIPIN-1, LIPIN-1α* or *LIPIN-1β* around 2-fold but it did not reach statistical differences compared to the control groups. In contrast, hepatocyte-specific HIF-1β knockout mice already had much higher basal expression of hepatic total *LIPIN-1*, *LIPIN-1α* and *LIPIN-1β* (almost elevated to 5–7 fold compared to wild type control groups). However, the ratio of *LIPIN-1β*/*LIPIN-1α* was not altered, and were not affected by Gao-binge treatment (**Fig. 5** **-E & F**). These results indicate that there was a persistent increased expression of hepatic *LIPIN-1* in hepatocyte-specific HIF-1β knockout mice. However, since the ratio of *LIPIN-1β*/*LIPIN-1α* was not altered, the impact of LIPIN-1 could be less important on the inhibitory effects against Gao-binge treatment-induced steatosis in hepatocyte-specific HIF-1β knockout mice.

### Changes in hepatic inflammation after Gao-binge treatment in wild type and HIF-1β hepatocyte-specific knockout mice

**Figure 6:**
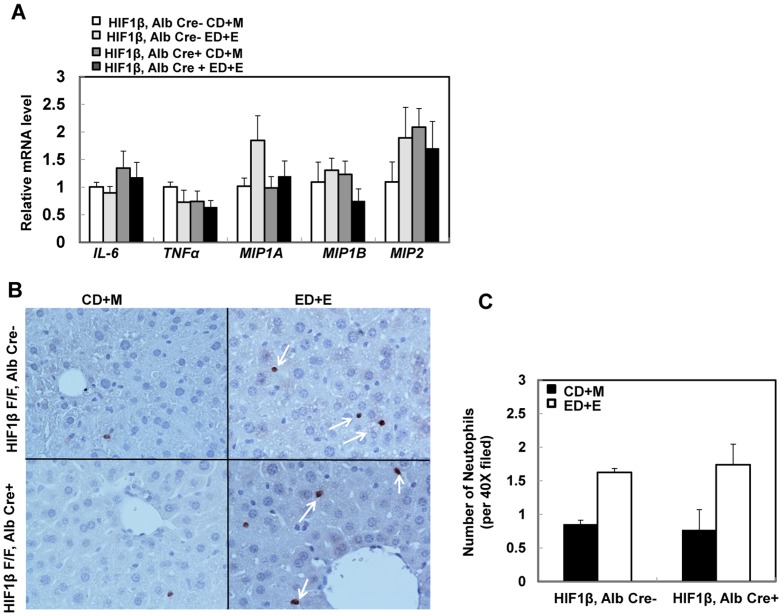
Changes in markers of hepatic inflammation in wild type and hepatocyte-specific HIF-1β knockout mice after Gao-binge treatment. Age matched male wild type and hepatocyte-specific HIF-1β knockout mice were subjected to Gao-binge treatment. Hepatic mRNA was isolated, and real-time RT-PCR was performed as described in the Materials and Methods (A). Data are presented as means ± SE (n = 3–5). Liver tissue was subjected to immunostaining for neutrophils using an anti-Ly6B antibody and representative images are shown in (B, 40x), and the number of neutrophils were quantified from 10 different fields of each mouse. (C). Data are presented as average number of neutrophils in each filed (means ± SE; n = 4).

One of the important features of Gao-binge treatment is increased inflammation in mouse livers compared to other alcohol mouse models (such as acute binge and chronic Lieber-DeCarlie alcohol diet) [1], [17], [28]. We next determined the hepatic expression of several inflammatory genes in wild type and hepatocyte-specific HIF-1β knockout mice after Gao-binge treatment. Gao-binge treatment did not induce significant changes on the hepatic expression of *MIP1A*, *MIP2*, *IL-6*, *TNF-α* or *MIP1B* in wild type mice. Except for a modest increase in basal expression of *MIP2* in hepatocyte-specific HIF-1β knockout mice, all other inflammatory genes that we assessed had minimal changes regardless of Gao-binge treatment compared to wild type control mice (**Fig. 6A**). Since it has been reported that Gao-binge treatment increased hepatic neutrophil infiltration, we next performed immunostaining for neutrophils using an anti-Ly6B antibody in mouse liver tissues after Gao-binge treatment. We found that Gao-binge treatment increased the number of hepatic neutrophils to almost 50% compared to control mice however there was no difference between wild type and hepatocyte-specific HIF-1β knockout mice (**Fig. 6B & C**). These data suggest that Gao-binge treatment may cause mild hepatic inflammation that does not require HIF-1β.

### Hepatic FoxO3a in hepatocyte-specific HIF-1β knockout mice may be associated with the protective effects against Gao-binge treatment-induced liver injury

**Figure 7:**
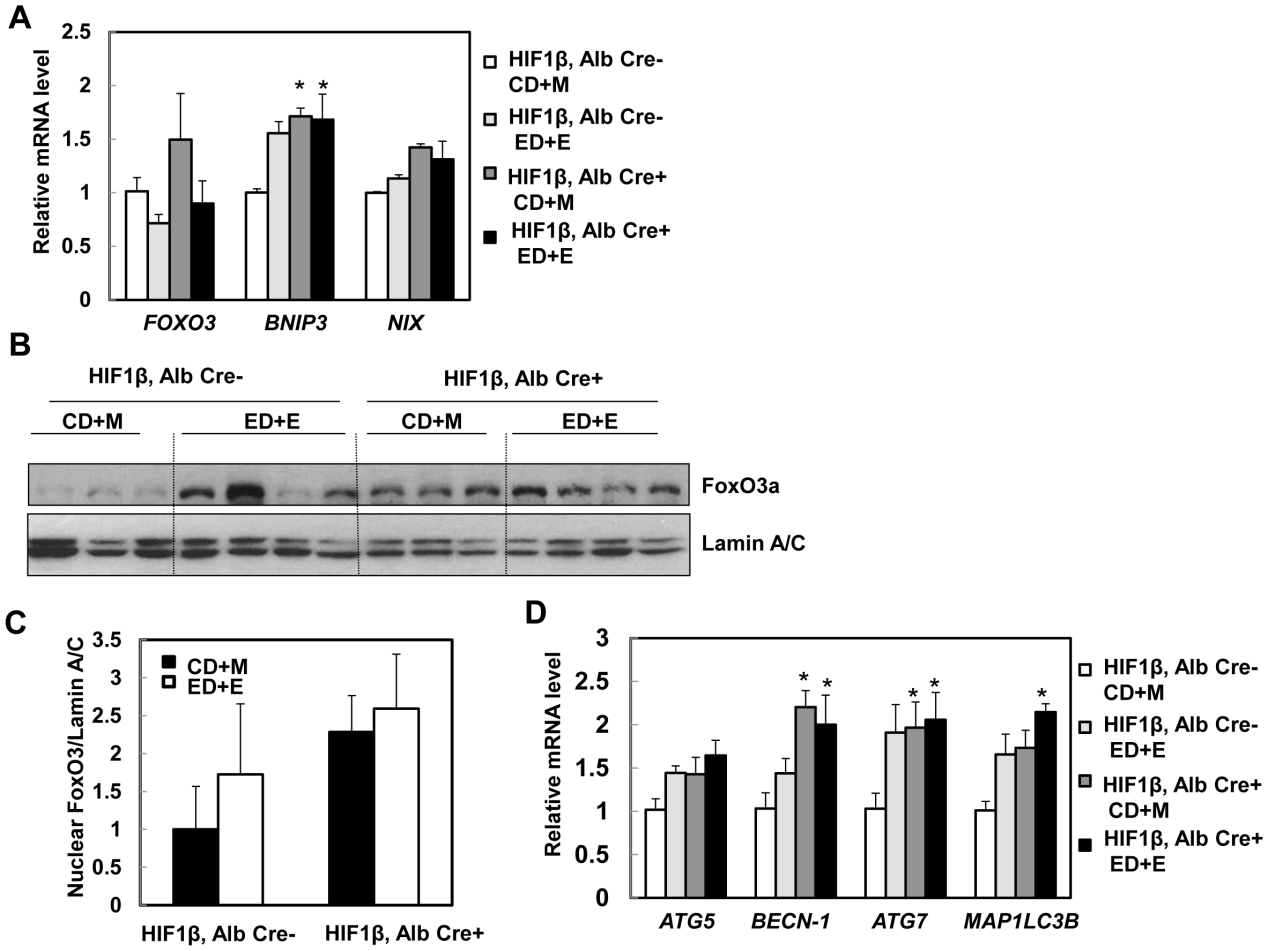
Changes of FoxO3a in hepatocyte-specific HIF-1β knockout mouse livers. Matched male wild type and hepatocyte-specific HIF-1β knockout mice were subjected to Gao-binge treatment. Hepatic mRNA was isolated, and real-time RT-PCR was performed as described in the Materials and Methods to quantify the expression of *FOXO3*, *BNIP3* and *NIX* (**A**). Data are presented as means ± SE (n = 3–5). * p<0.05, vs HIF-1β CD+M group. One way ANOVA with Scheffé's post hoc test. Mice were treated as in (**A**), mouse liver nuclear fractions were prepared as described in the Materials and Methods followed by western blot analysis (**B**) and densitometry analysis (**C**). Data are presented as means ± SE (n = 3). (**D**) Mice were treated as in (**A**), hepatic mRNA was isolated, and real-time RT-PCR was performed as described in the Materials and Methods to quantify autophagy-related genes. Data are presented as means ± SE (n = 3–5). * p<0.05; * p<0.05, vs HIF-1β CD+M group. One way ANOVA with Scheffé's post hoc test.

**Figure 8:**
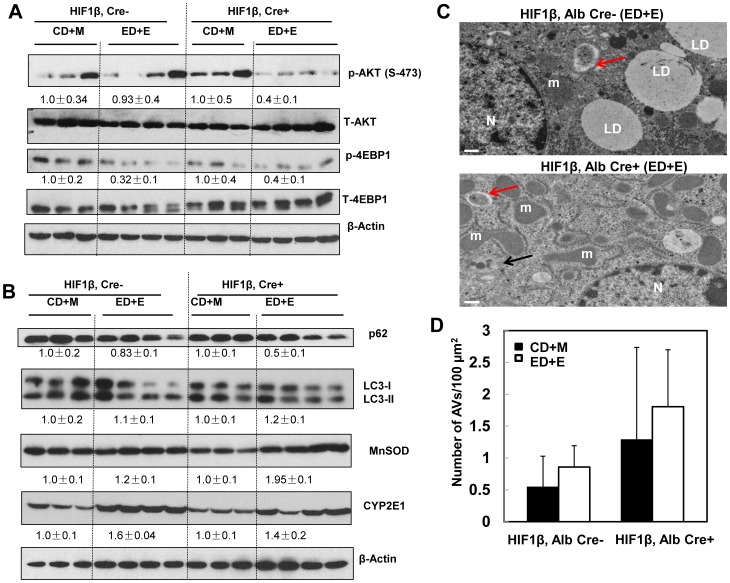
Inhibition of AKT and mTOR and induction of autophagy in hepatocyte-specific HIF-1β knockout mouse livers after Gao-binge treatment. Age matched male wild type and hepatocyte-specific HIF-1β knockout mice were subjected to Gao-binge treatment. (A & B) Total liver lysates were subjected to western blot analysis for the indicated proteins. Densitometry was performed and data are presented as a ratio of CD+M control group (n = 3–4). Representative EM images are shown in (C). M: Mitochondria; N: Nuclei; LD: lipid droplet; Bar: 500 nm. Red arrows: autophagosomes, black arrows: autolysosomes. (D) The number of autophagic vacuoles (AVs, including both autophagosomes and autolysosomes) per 100 µm^2^ cytosol was quantified, and data are presented as means ± SE (more than 15 cell sections).

We next quantified hepatic levels of BNIP3 and NIX in wild type and hepatocyte-specific HIF-1β knockout mice after Gao-binge treatment. We found that Gao-binge treatment slightly increased hepatic expression of *BNIP3* and *NIX* in HIF-1β wild type mice (increase 0.5 and 0.2 fold respectively) (**Fig. 7A**). In contrast to our *in vitro* findings (**Fig. 2B**), however, hepatic expression of *BNIP3* did not decrease but rather increased in hepatocyte-specific HIF-1β knockout mice regardless of Gao-binge treatment (**Fig. 7A**). The expression of hepatic *NIX* was also slightly increased at baseline and in alcohol-treated hepatocyte-specific HIF-1β knockout mice but did not reach statistical difference (**Fig. 7A**). It should be noted that Gao-bine treatment did not lead significantly increased the expression levels of BNIP3 and NIX in HIF-1β wild type mice compared to acute alcohol treated wild type mice. The difference of the mouse strains and the Gao-binge vs acute alcohol treatment may contribute to these different observations. In addition to HIF, FoxO3a, another transcriptional factor, also regulates hepatic expression of *BNIP3* and *NIX* [29], [30]. We thus tested the hypothesis that FoxO3a is activated in hepatocyte-specific HIF-1β knockout mouse livers and that this could serve as a compensatory mechanism for the loss of HIF-1β. Indeed, there was an increase of hepatic *FOXO3* expression in hepatocyte-specific HIF-1β knockout mice compared to wild type mice although this did not reach statistical difference. Gao-binge treatment slightly decreased hepatic expression of *FOXO3* in both wild type and hepatocyte-specific HIF-1β knockout mice (**Fig. 7A**). FoxO3a is mainly regulated at the post-translational level and de-phosphorylated FoxO3a is mainly retained in the nucleus where it regulates gene expression [31], [32]. We found that there was an increase in basal hepatic nuclear FoxO3a levels in hepatocyte-specific HIF-1β knockout mice compared to wild type mice. Gao-binge treatment increased nuclear FoxO3a levels in wild type mice but not in hepatocyte-specific HIF-1β knockout mice likely due to the saturation of nuclear FoxO3a in hepatocyte-specific HIF-1β knockout mice (**Fig. 7B & C**). Consistent with these findings, we also found that hepatic expression of *ATG5, BECN-1*, *ATG7* and *MAP1/LC3B*, which are regulated by FoxO3a, were increased in Gao-binge-treated wild type mice. Consistent with the increased FoxO3a activity in hepatocyte-specific HIF-1β knockout mouse livers, basal levels of *BECN-1 and*, *ATG7* were already significantly higher in the hepatocyte-specific HIF-1β knockout mice, which did not change after Gao-binge treatment (**Fig. 7D**). The basal expressions of other FoxO3a-mediated autophagy related genes were also higher in hepatocyte-specific HIF-1β knockout mouse livers compared to wild type mice but did not reach statistical difference. AKT is one of the major kinases that phosphorylates FoxO3a resulting in reduced nuclear FoxO3a retention [30], [31]. We found that Gao-binge treatment markedly reduced levels of phosphorylated AKT in hepatocyte-specific HIF-1β knockout mice but that it only had mild effects in wild type mice (**Fig. 8A**). Interestingly, levels of phosphorylated 4EBP-1, one of the mammalian target of rapamycin (mTOR) substrates, were markedly decreased in both wild type and hepatocyte-specific HIF-1β knockout mice after Gao-binge treatment (**Fig. 8A**). We recently demonstrated that activation of FoxO3a promotes hepatic autophagy and protects against acute ethanol-induced liver injury [19]. Indeed, we found that hepatic p62, which is normally degraded in the autolysosomes as a result of increased autophagic flux, were markedly decreased in hepatocyte-specific HIF-1β knockout mice but only mildly decreased in wild type mice following Gao-binge treatment (**Fig. 8B**). We did not find significant changes in the levels of LC3-II in both wild type and hepatocyte-specific HIF-1β knockout mice. It is likely that LC3-II could be degraded in the autolysosomes to offset the possible increased autophagosome formation induced by ethanol treatment (**Fig. 8B**). Moreover, we also found that MnSOD, another FoxO3a target gene, was markedly elevated in hepatocyte-specific HIF-1β knockout mouse livers when compared to wild type mice, whereas induction of CYP2E1 was very comparable in both wild type and hepatocyte-specific HIF-1β knockout mice after Gao-binge treatment (**Fig. 8B**). In line with our western blot analysis, EM studies revealed that Gao-binge treatment slightly increased the number of autophagosome/autolysosomes (AVs) in wild type mouse livers. The number of AVs was increased by two fold in hepatocyte-specific HIF-1β knockout mouse livers compared to wild type mice at baseline, and was further increased by Gao-binge treatment in hepatocyte-specific HIF-1β knockout mouse livers although differences were not significant (**Fig. 8C & D**).

**Figure 9:**
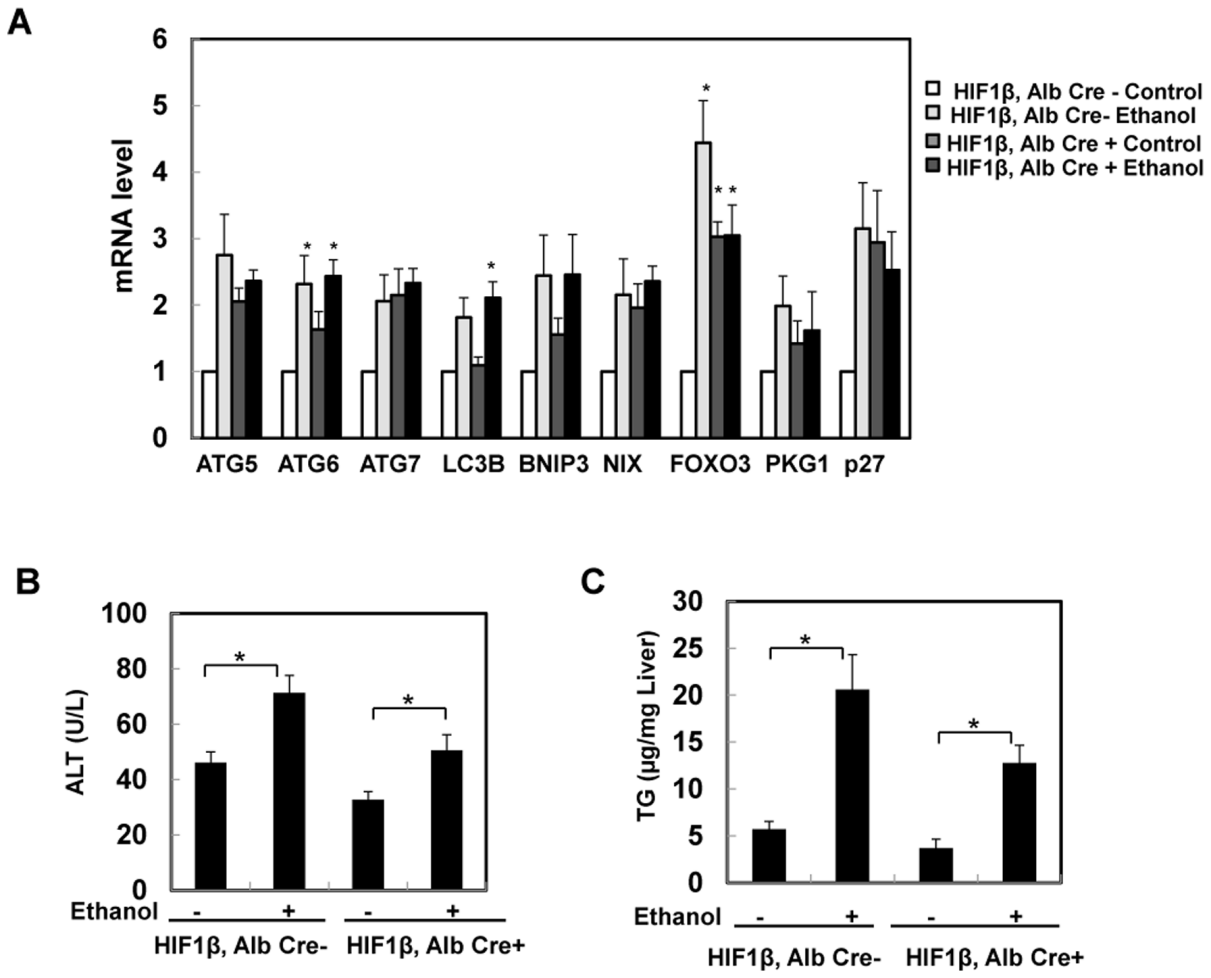
Hepatocyte-specific HIF-1β knockout mice were resistant to acute ethanol treatment-induced steatosis and liver injury. Age matched male wild type and hepatocyte-specific HIF-1β knockout mice were subjected to acute ethanol treatment for 16 hours. Hepatic mRNA was isolated, and real-time RT-PCR was performed as described in the Materials and Methods (**A**). Data are presented as means ± SE (n = 3–5). Serum ALT (**A**) and hepatic TG (**B**) were measured. Data are presented as means ± SE (n = 3–5). * p<0.05, vs HIF-1β, Alb Cre- control group or HIF-1β, Alb Cre+ control group, One way ANOVA with Scheffé's post hoc test.

We next determined whether hepatocyte-specific HIF-1β knockout mice were also resistant to acute alcohol-induced liver injury. We previously reported that acute ethanol treatment activates FoxO3a resulting in increased hepatic expression of autophagy genes in C57Bl/6 mice [19]. Consistent with our previous report, we found that acute ethanol treatment increased the expression of autophagy related genes (*ATG5, ATG6, ATG7, LC3B*, *BNIP3* and *NIX*) and FoxO3a target genes (*FOXO3, PKG1* and *p27*) compared to control group. There was almost a 2-fold increase in all the genes that we assessed although only ATG6 and FOXO3 showed statistical differences compared to the control group. Interestingly, basal expression of all genes we assessed was nearly 2-fold higher in hepatocyte-specific HIF-1β knockout mice when compared to the wild type control mice at baseline, although, there were no significant change after acute ethanol treatment in hepatocyte-specific HIF-1β knockout mice (**Fig. 9A**). More importantly, hepatocyte-specific HIF-1β knockout mice had decreased serum ALT and hepatic TG levels after acute ethanol treatment compared to wild type mice although these changes were not statistically different compared to acute ethanol-treated wild type mice (**Fig. 9B & C**). Taken together, these results suggest that hepatocyte-specific HIF-1β knockout mice may have increased FoxO3a activation and autophagy, which may be associated with the decreased steatosis and liver injury after Gao-binge and acute ethanol treatment in hepatocyte-specific HIF-1β knockout mice.

## Discussion

Previous studies showed that chronic alcohol consumption produces liver hypoxia and activates the transcription factor HIF-1α [3], [6], [7], [25]. However, conflicting results have been reported regarding the role of HIF-1α in chronic alcohol-induced liver injury despite a similar Lieber-DeCarli liquid diet in both studies [6], [7]. The reasons for this discrepancy are not clear, but different control animals were used in these studies (normal C57BL/6 mice or matched HIF-1α Flox/Flox Cre negative mice). Moreover, differences in housing environments may have also contributed to the conflicting observations [8]. There are three HIF-α subunits (HIF1-α, HIF-2α and HIF-3α) in the liver, and all of them must dimerize with HIF-1β to trigger transcription of target genes [33]. It has been reported that knockout one of the α subunits can lead to a compensatory activation of other α subunits [9], and this possibility has not been considered in the two previous conflicting studies on ALD. In the present study, we used hepatocyte-specific HIF-1β knockout mice, which would eliminate the compensatory effects of a single knockout of one α subunit. We found that hepatocyte-specific HIF-1β knockout mice were resistant to alcohol-induced steatosis and liver injury following Gao-binge treatment. Hepatocyte-specific HIF-1β knockout mice had increased FoxO3a activation that might result in FoxO3a-mediated autophagy induction and increased antioxidant capacity (MnSOD), all these events may be associated with the decreased alcohol-induced steatosis and liver injury.

We found that deletion of HIF-1β in mouse liver protected against alcohol-induced fatty liver/steatosis. Although steatosis has been thought to be a reversible and benign condition in ALD, chronic steatosis may render the liver more susceptible to the development of alcoholic hepatitis, fibrosis or cirrhosis. Thus, understanding the mechanisms that regulate alcohol-induced steatosois and control hepatic triglyceride accumulation could help to ameliorate ALD. Alcohol consumption increases the uptake of fatty acids into the liver and increases fatty acid and triglyceride synthesis, whereas it inhibits the secretion of hepatic VLDL and impairs mitochondrial function possibly reducing fatty acid oxidation [27], [34], [35], [36], [37], [38], [39], [40], all of these contribute to alcoholic steatosis although it seems that the predominant mechanisms differ between experimental ALD animal models. We found that Gao-binge treatment did not increase the expression of lipogenesis genes but instead caused a decrease in expression of several lipogenesis genes and an increase in *DEC2* expression that negatively regulates SREBP-1c. These data seem to suggest that *de novo* lipogenesis may not be a major contributor for hepatic steatosis induced by Gao-binge treatment. Basal hepatic expression of *CPT1* and *ACOX1*, two important genes regulating mitochondrial and peroxisosomal fatty acid oxidation, were much higher in hepatocyte-specific HIF-1β knockout mice. These results suggest that it is possible that increased burning of fat in hepatocyte-specific HIF-1β knockout mice may help to ameliorate steatosis induced by Gao-binge treatment. Future works are needed to directly assess mitochondrial and peroxisomal fatty acid oxidation after alcohol treatment in wild type and hepatocyte-specific HIF-1β knockout mice. In addition to lipogenesis and fatty acid oxidation, recent evidence suggest that autophagy may help to remove excess intracellular lipid droplets (a term referred to as lipophagy) [41], [42]. Indeed, we have previously demonstrated that induction of autophagy protects against acute ethanol-induced steatosis and liver injury in mice [10]. Accumulating evidence now supports that hypoxia can induce autophagy in cultured cells and mouse tissues [43], [44], [45]. Mechanistically, both HIF-dependent and –independent mechanisms have been shown to contribute to hypoxia-induced autophagy. The HIF-dependent mechanism mainly relies on the transcriptional up-regulation of two atypical BH3-domain containing proteins, BNIP3 and NIX. BNIP3 and NIX can compete with Beclin-1, another atypical BH3-domain containing protein that is essential for autophagy induction by promoting Vps34 kinase activity for the autophagosomal vesicle nucleation, for binding with Bcl-2/Bcl-xL that are negative regulators for autophagy. Increased levels of BNIP3 and NIX dissociate Beclin 1 from Bcl-2/Bcl-xL to induce autophagy [46]. However, HIF-dependent transcriptional activation is not always required for autophagy. Under severe hypoxic conditions, Amp-activated protein kinase (AMPK)-mediated mTOR suppression as well as endoplasmic reticulum stress-mediated unfolded protein response can also trigger autophagy independent of HIF [46], [47]. In primary cultured hepatocytes, we found that ethanol treatment still induced autophagic flux in HIF-1β knockout hepatocytes. More intriguingly, hepatocyte-specific HIF-1β knockout mice seemed to have higher basal and alcohol-induced autophagy than wild type mice in the mouse livers. These results suggest that ethanol may activate HIF- independent pathways to induce autophagy. We found that the levels of phosphorylated 4EBP-1, which is normally used to assess mTOR activity, were decreased in both wild type and hepatocyte-specific HIF-1β knockout mouse livers after Gao-binge treatment, suggesting that decreased mTOR activity could be one of the mechanisms for autophagy induction after Gao-binge treatment in hepatocyte-specific HIF-1β knockout mice.

In addition to HIF, FoxO family proteins can regulate autophagy by at least three distinct mechanisms: direct transcriptional up-regulation of autophagy-related genes including BNIP3 and NIX [29], [30], modulation of intracellular glutamine levels [48], and direct interaction with ATG7 independent of transcriptional activity [49]. Recent studies from our laboratory and others suggested that FoxO3a plays a role in reducing alcohol-induced steatosis and hepatotoxicity [19], [50], [51]. Using an acute ethanol binge model, we demonstrated that acute ethanol activates FoxO3a-mediated transcription for multiple autophagy related genes and FoxO3 knockout mice have decreased hepatic autophagy and exacerbated acute ethanol-induced liver injury [19]. Consistent with our findings, FoxO3 knockout mice fed the Lieber-DeCarli alcohol diet for 3 weeks developed more severe steatosis, inflammation and liver injury compared to wild type mice likely due to decreased expression of MnSOD, another target gene of FoxO3a that attenuates oxidative stress [51]. We found that there was increased hepatic FoxO3a activation in hepatocyte-specific HIF-1β knockout mice at the basal level and after Gao-binge treatment resulting in increased hepatic autophagy. FoxO3a activation is mainly regulated by post-translational modifications, including phosphorylation, acetylation, methylation and ubiquitination [50]. AKT-mediated phosphorylation of FoxO3a causes its nuclear exclusion which inactivates FoxO3a. We found that there was decreased AKT phosphorylation after Gao-binge treatment in hepatocyte-specific HIF-1β knockout mouse livers. We also found that there were increased nuclear FoxO3a levels as well as increased expression of hepatic autophagy related genes and MnSOD in hepatocyte-specific HIF-1β knockout mice after Gao-binge treatment. As a result, we also found increased autophagy induction in hepatocyte-specific HIF-1β knockout mouse livers. It should be noted that our *in vivo* findings were somewhat different from the findings in ethanol- treated primary cultured mouse hepatocytes, in which we found that the expression of BNIP3 and NIX was much lower in HIF-1β knockout hepatocyte whereas their autophagy status was quite comparable to wild type hepatocytes. Under *in vivo* conditions, hepatocytes were exposed to a variety of endocrine and exocrine nutritional and growth factors such as insulin, which could be altered due to the chronic deletion of HIF-1β. All these factors may affect AKT and FoxO3a activation, which might be absent in the culture conditions. While it is most likely that increased autophagy may offer the protection against Gao-binge treatment-induced steatosis and liver injury in hepatocyte-specific HIF-1β knockout mice, future work is needed to further confirm the role of FoxO3a by generating FoxO3a and HIF-1β double knockout mice.

In conclusion, we have demonstrated that hepatocyte-specific HIF-1β knockout mice were resistant to alcohol-induced steatosis and liver injury in the recently established Gao-binge model. This protection was associated with activation of FoxO3a-mediated hepatic autophagy.

Our findings provided evidence to support the possible detrimental role of alcohol-induced HIF-1 activation in ALD as previously reported [7], and may also help to further clarify previous conflicting findings using hepatocyte-specific HIF-1α knockout mice.
